# Utilisation Trend of Long-Acting Insulin Analogues including Biosimilars across Europe: Findings and Implications

**DOI:** 10.1155/2021/9996193

**Published:** 2021-10-11

**Authors:** Brian Godman, Magdalene Wladysiuk, Stuart McTaggart, Amanj Kurdi, Eleonora Allocati, Mihajlo Jakovljevic, Francis Kalemeera, Iris Hoxha, Anna Nachtnebel, Robert Sauermann, Manfred Hinteregger, Vanda Marković-Peković, Biljana Tubic, Guenka Petrova, Konstantin Tachkov, Juraj Slabý, Radka Nejezchlebova, Iva Selke Krulichová, Ott Laius, Gisbert Selke, Irene Langner, András Harsanyi, András Inotai, Arianit Jakupi, Svens Henkuzens, Kristina Garuolienė, Jolanta Gulbinovič, Patricia Vella Bonanno, Jakub Rutkowski, Skule Ingeberg, Øyvind Melien, Ileana Mardare, Jurij Fürst, Sean MacBride-Stewart, Carol Holmes, Caridad Pontes, Corinne Zara, Marta Turu Pedrola, Mikael Hoffmann, Vasileios Kourafalos, Alice Pisana, Rita Banzi, Stephen Campbell, Bjorn Wettermark

**Affiliations:** ^1^Strathclyde Institute of Pharmacy and Biomedical Sciences, University of Strathclyde, Glasgow G4 0RE, UK; ^2^Division of Public Health Pharmacy and Management, School of Pharmacy, Sefako Makgatho Health Sciences University, Pretoria, South Africa; ^3^School of Pharmaceutical Sciences, Universiti Sains Malaysia, Penang, Malaysia; ^4^Chair of Epidemiology and Preventive Medicine, Medical College, Jagiellonian University, Krakow, Poland; ^5^HTA Consulting, Starowiślna Str. 17/3, 31-038 Krakow, Poland; ^6^Public Health Scotland, Gyle Square, 1 South Gyle Crescent, Edinburgh, UK; ^7^Department of Pharmacology, College of Pharmacy, Hawler Medical University, Erbil, Iraq; ^8^Istituto di Ricerche Farmacologiche ‘Mario Negri' IRCCS, Milan, Italy; ^9^Department of Global Health Economics and Policy, University of Kragujevac, Kragujevac, Serbia; ^10^Institute of Comparative Economic Studies, Faculty of Economics, Hosei University Tokyo, Tokyo, Japan; ^11^Department of Pharmacy Practice and Policy, Faculty of Health Sciences, University of Namibia, Windhoek, Namibia; ^12^Department of Pharmacy, Faculty of Medicine, University of Medicine, Tirana, Albania; ^13^Dachverband der Österreichischen Sozialversicherungen, Kundmanngasse 21, AT-1030 Vienna, Austria; ^14^Faculty of Medicine, Department of Social Pharmacy, University of Banja Luka, Banja Luka, Bosnia and Herzegovina; ^15^Faculty of Medicine, Department of Medicinal Chemistry, University of Banja Luka, Banja Luka, Bosnia and Herzegovina; ^16^Agency for Medicinal Product and Medical Devices of Bosnia and Herzegovina, 78000 Banja Luka, Bosnia and Herzegovina; ^17^Faculty of Pharmacy, Department of Social Pharmacy and Pharmacoeconomics, Medical University of Sofia, Sofia, Bulgaria; ^18^State Institute for Drug Control, Prague, Czech Republic; ^19^Department of Medical Biophysics, Faculty of Medicine in Hradec Králové, Charles University, Simkova 870, 500 03 Hradec Králové, Czech Republic; ^20^State Agency of Medicines, Nooruse 1, 50411 Tartu, Estonia; ^21^Wissenschaftliches Institut der AOK (WIdO), Rosenthaler Straße 31, 10178 Berlin, Germany; ^22^Department of Health Policy and Health Economics, Eotvos Lorand University, Budapest, Hungary; ^23^Syreon Research Institute and Semmelweis University, Center of Health Technology Assessment, Budapest, Hungary; ^24^Faculty of Pharmacy, UBT Higher Education Institute, Pristina, Kosovo; ^25^Independent Consultant, Riga, Latvia; ^26^Department of Pathology, Forensic Medicine and Pharmacology, Institute of Biomedical Sciences, Faculty of Medicine, Vilnius University, Vilnius, Lithuania; ^27^Department of Health Services Management, University of Malta, Valletta, Malta; ^28^Medicines Committee, Oslo University Hospitals, Oslo, Norway; ^29^Faculty of Medicine, Public Health and Management Department, “Carol Davila” University of Medicine and Pharmacy Bucharest, 050463 Bucharest, Romania; ^30^Health Insurance Institute, Miklosiceva 24, SI-1507 Ljubljana, Slovenia; ^31^Pharmacy Services, Greater Glasgow and Clyde (NHS GGC), Glasgow, UK; ^32^NHS Lothian, Edinburgh, UK; ^33^Drug Department, Catalan Health Service, Gran Via de les Corts Catalanes, 08007 Barcelona, Spain; ^34^Department of Pharmacology, Therapeutics and Toxicology, Universitat Autònoma de Barcelona, Barcelona, Spain; ^35^NEPI-Nätverk för läkemedelsepidemiologi, Stockholm, Sweden; ^36^National Organization for the Provision of Healthcare Services (EOPYY), Athens, Greece; ^37^Department of Global Public Health, Karolinska Institutet, Stockholm, Sweden; ^38^Centre for Primary Care and Health Services Research, School of Health Sciences, University of Manchester, Manchester M13 9PL, UK; ^39^NIHR Greater Manchester Patient Safety Translational Research Centre, School of Health Sciences, University of Manchester, Manchester, UK; ^40^Department of Pharmacy, Disciplinary Domain of Medicine and Pharmacy, Uppsala University, Uppsala, Sweden

## Abstract

**Background:**

Diabetes mellitus rates and associated costs continue to rise across Europe enhancing health authority focus on its management. The risk of complications is enhanced by poor glycaemic control, with long-acting insulin analogues developed to reduce hypoglycaemia and improve patient convenience. There are concerns though with their considerably higher costs, but moderated by reductions in complications and associated costs. Biosimilars can help further reduce costs. However, to date, price reductions for biosimilar insulin glargine appear limited. In addition, the originator company has switched promotional efforts to more concentrated patented formulations to reduce the impact of biosimilars. There are also concerns with different devices between the manufacturers. As a result, there is a need to assess current utilisation rates for insulins, especially long-acting insulin analogues and biosimilars, and the rationale for patterns seen, among multiple European countries to provide future direction. *Methodology*. Health authority databases are examined to assess utilisation and expenditure patterns for insulins, including biosimilar insulin glargine. Explanations for patterns seen were provided by senior-level personnel.

**Results:**

Typically increasing use of long-acting insulin analogues across Europe including both Western and Central and Eastern European countries reflects perceived patient benefits despite higher prices. However, activities by the originator company to switch patients to more concentrated insulin glargine coupled with lowering prices towards biosimilars have limited biosimilar uptake, with biosimilars not currently launched in a minority of European countries. A number of activities were identified to address this. Enhancing the attractiveness of the biosimilar insulin market is essential to encourage other biosimilar manufacturers to enter the market as more long-acting insulin analogues lose their patents to benefit all key stakeholder groups.

**Conclusions:**

There are concerns with the availability and use of insulin glargine biosimilars among European countries despite lower costs. This can be addressed.

## 1. Introduction

Global expenditure on medicines is envisaged to reach US$1.5 trillion by 2023 enhanced by growing prevalence rates for noncommunicable diseases (NCDs) [1, 2]. This is a concern among European countries given their desire to retain universal healthcare as a core principle as well as limit out-of-pocket expenditures especially among citizens with low income [3–6]. Currently across Europe, approximately one-fifth of health spending is paid for out of pocket, with a higher proportion among those with low income potentially leading to catastrophic consequences [4].

One NCD of increasing priority is diabetes mellitus, where prevalence rates grew to 463 million people worldwide in 2019 [7, 8]. In Europe, approximately 59 million people are currently estimated to have diabetes, with this number predicted to rise to 68 million by 2045 [9]. Whilst the majority of these patients will have type 2 diabetes (T2DM), up to 30% or more of patients with diabetes require insulin to help control HbA1c levels [10–13].

As a result of growing prevalence rates, the global economic burden of diabetes is envisaged to be as high as 2.2% of Gross Domestic Product (GDP) by 2030 [14], supported by GDP growth rates worldwide across many countries including developing countries [15, 16]. The economic impact of diabetes is enhanced by the cost of the complications including complications arising from hypoglycaemia [9, 13, 17, 18]. This is important with estimated rates of hypoglycaemia up to 3.5–3.6 events/month among patients with type 1 diabetes (T1DM) and 2.2–3.7 among those with type 2 diabetes (T2DM) [19–23], with some authors finding that rates of hypoglycaemia may be similar for T2DM patients taking insulins for >5years [13]. In addition, the serious consequences of hypoglycaemia may turn out to be greater in T2DM patients, particularly regarding the effects on the cardiovascular system [13]. Overall, diabetes is among the leading causes of nontraumatic lower extremity amputation and blindness worldwide, with patients with diabetes also at greater risk of cardiovascular disease [7, 24–26]. In view of this, it is important that patients with diabetes should be carefully managed, which includes reducing the risk of hypoglycaemia [13].

Long-acting insulin analogues were specifically developed to lower the risk of hypoglycaemia in patients with diabetes requiring insulin, especially nocturnal hypoglycaemia, as well as improve patient convenience through reducing the number of injections thereby enhancing adherence rates, which is a continuing concern with insulin [7, 9, 27–30]. There is still controversy though regarding the level of benefit seen with long-acting insulin analogues versus NPH and other insulins [31–33]. However, a recent systematic review and network meta-analysis suggests that long-acting insulin analogues were superior to intermediate-acting insulins in key areas including major, serious, and nocturnal hypoglycaemia [34]. Having said this, the perceived patient benefits of long-acting insulin analogues are potentially reflected by their usage now typically exceeding that of human insulins in upper-middle and high-income countries as well as growing in lower middle-income countries including Bangladesh [35–37]. In addition, global expenditure on insulin glargine was already US$3.88 billion in 2018 out of a total market of US$24million and envisaged to potentially reach as high as US$9.26 billion by 2025 helped by growing sales of Toujeo® 300 IU/ml [38, 39]. Expenditure on insulin detemir was US$2.7 billion in 2015, growing at 7.5% per year [40], with sales of insulin degludec also growing with studies demonstrating their improved effectiveness and cost-effectiveness versus other long-acting insulin analogues [41–45].

However, there are concerns with the high costs of long-acting insulin analogues compared to Neutral Protamine Hagedorn (NPH) and other insulins [31, 35, 46]. This is not universal though with published studies showing that the higher acquisition costs of long-acting insulin analogues can be fully or partially offset by savings from averted costs of hypoglycaemia and other diabetes-associated complications [47–51].

Biosimilars are a potential way forward to reduce the cost of long-acting insulin analogues building on the appreciable price reductions seen with biosimilars to treat rheumatoid arthritis [52–56]. In addition, a number of published studies have now demonstrated similar effectiveness and safety between the originator and biosimilar long-acting insulin analogues [57–61]. However, potential savings from biosimilar insulin glargine can be limited in practice, potentially accentuated by the dominance of three companies currently controlling 96% of the global insulin market by volume and 99% by value discouraging competition [36, 46]. We have seen this in the United Kingdom with limited price differences with Semglee® (biosimilar insulin glargine 100 IU/ml) currently priced only 20% below the originator price and only 15% below the price of Abasaglar® (another biosimilar insulin glargine) [62]. Alongside this, there are concerns with increased rates of hypoglycaemia if patients are switched between different formulations of insulin glargine 100 IU/ml with different devices without full patient education [63–65]. These limited price differences were also seen in a recent study by Ewen et al. where median biosimilar prices for insulin glargine across lower- and middle-income (LMIC) countries ranged from 2% to 25% below originator prices, and sometimes biosimilar prices were higher in private pharmacies [35]. However, this was not the case in a recent study in Bangladesh with appreciable price reductions for biosimilar insulin glargine enhanced by competition between manufacturers [37]. WHO prequalification should also enhance competition leading to lower prices for biosimilar long-acting insulin analogues [66]. This is welcomed since limited price reductions for the biosimilar analogues can easily be matched by the originator company to protect its market given envisaged low cost of goods apart from insulin detemir [46, 67]. As a result, the attractiveness of the European long-acting insulin analogue market for biosimilar manufacturers could be potentially reduced, and thereby, possible competition leading to lower prices.

Concerns regarding the different devices between the originator and biosimilars may well have resulted in the low use of insulin glargine biosimilars (9%) among diabetologists in the UK in 2017 further limiting the attractiveness of the long-acting insulin biosimilar market [68]. However, this is not universal with some commissioning groups in England achieving utilisation rates of 53.3% for biosimilar insulin glargine in December 2018 versus total insulin glargine [69].

Other activities to reduce the attractiveness of the long-acting insulin analogue market for biosimilar manufacturers include the originator company launching more concentrated patented formulations to enhance patient convenience and potentially further reduce rates of hypoglycaemia, i.e., a 300 IU/ml formulation of insulin glargine (Gla-300) [41, 70–75]. Having said this, other researchers have found no difference in effectiveness between the different strengths of insulin glargine and concerns with possible underdosing with the 300 IU/ml formulation [76]. These “evergreening” activities by the originator company to preserve its market share in the face of potential competition are similar to the launch of different devices for the treatment of asthma to try and improve adherence rates and protect sales as well as the development of longer-acting oral formulations and intramuscular formulations of atypical antipsychotics to improve compliance and reduce recurrences [77–80]. Such company activities are also seen in other disease areas. These include the launch of esomeprazole versus omeprazole, escitalopram versus citalopram, and pregabalin versus gabapentin [81–85]. We are aware of prescribing restrictions for Gla-300 in some of the European countries [86]; however, this is not universal, and sales are growing especially with publications suggesting improved cost-effectiveness versus 100 IU/ml formulations [41].

Consequently, in view of the current controversies and issues surrounding the use of long-acting insulin analogues as well as the biosimilars, we believe that there is a need to assess current utilisation and expenditure patterns for the long-acting insulin analogues including biosimilars across Europe and the rationale for any patterns seen. The findings can be used by health authorities across Europe to enhance the use of biosimilar long-acting insulin analogues where pertinent to limit the budget impact of increasing the number of patients with diabetes across Europe including those requiring insulins. This will be important to preserve universal access especially post-COVID-19 with its resultant impact on available resources coupled with increases in patients with NCDs and their complications as a result of lockdown and other measures [87, 88].

## 2. Materials and Methods

We included a range of European countries incorporating both Western as well as Central and Eastern European (CEE) countries covering a range of geographies, epidemiology, and economic power in terms of GDP per population. This is similar to other studies conducted across Europe [89–91]. We particularly wanted to include CEE countries since there has been appreciably lower use of biologicals in these countries versus Western European countries due to issues of cost and affordability [92–96].

We typically used reimbursed data from heath authority and health insurance company databases from 2014 or later until 2020 when assessing utilisation and pricing patterns for the different insulin preparations. These were supplied by coauthors in each country since the content of these databases are typically not publicly available. This is different to studies by Beran et al. and Ewen and colleagues who use a wide variety of sources when computing cost data [35, 46, 97]. This is because the perspective of this paper is a health authority one; consequently, we concentrated on their databases. These databases are also seen as robust, and they are regularly audited [89, 98, 99]. Consequently, health authority data is seen as a reliable source for comparing and contrasting utilisation and expenditure patterns across countries [98]. We principally centred on insulin glargine as this is the only biosimilar insulin currently available across Europe at the time of the study.

Utilisation data was broken down into Defined Daily Doses (DDDs). This is because DDDs are seen as a key standard for comparing utilisation patterns across countries especially if there are different pack sizes and strengths between countries [100–102]. We acknowledge that some published studies have suggested that DDDs may understate the amount of insulin that patients prescribed 300 IU/ml insulin glargine receive versus those prescribed 100 IU/ml formulation; however, others have not seen this [74, 103]. We have used this approach before in multiple publications when assessing utilisation and expenditure patterns across disease areas and countries [89–91, 104–107].

Expenditure data was principally reimbursed data since, as mentioned, the perspective of this paper is a health authority one. In a minority of situations, we also used total expenditure where it proved difficult to break expenditures down into the individual components. This again is in line with previous publications [89–91, 104–108]. Expenditure data remained where relevant in the local currency as we were principally interested in percentage differences in costs over time between the originator and biosimilars, as well as price reductions over time, rather than absolute levels and without any influence from currency fluctuations.

Utilisation and expenditure data on insulin glargine was further broken down into the different formulations, e.g., different 100 IU/ml formulations, as well as for the 300 IU/ml formulation (Gla-300) since, as mentioned, we were aware that the parent company had been switching its promotional activities towards the patented 300 IU/ml formulation in recent years to protect its market and help deter biosimilar manufacturers.

We combined the information from over 20 European countries and regions to provide the following datasets for comparisons:

- Utilisation of long-acting insulin analogues as a percentage of total insulin utilisation based on DDDs
- Expenditure on long-acting insulin analogues as a percentage of total insulin expenditure based on local currencies
- Utilisation of biosimilar insulin glargine (100 IU/ml) as a percentage of total insulin glargine (100 IU/ml) again based on DDDs
- Utilisation of insulin glargine 300 IU/ml as a percentage of total insulin glargine again based on DDDs
- Cost/DDD for both originator and biosimilar insulin glargine (100 IU/ml) over time with the data subsequently used to track price changes over time

The information on utilisation and expenditure patterns was supplemented by feedback from the coauthors regarding the patterns seen in their countries to provide future guidance. The senior-level coauthors also contributed to discussions regarding potential next steps to enhance future savings from increased utilisation of biosimilars based on their considerable experience in this area. We have adopted similar approaches before to provide future guidance in this and other areas [55, 89, 104, 109–113].

We did not seek ethical approval as we were not dealing with patients. This is in line with national legislation and institutional guidelines as well as multiple previous papers conducted by the coauthors in other disease areas and situations [89, 104, 105, 114–116].

## 3. Results

### 3.1. Utilisation for the Different Insulin Preparations Over Time

**Figure 1:**
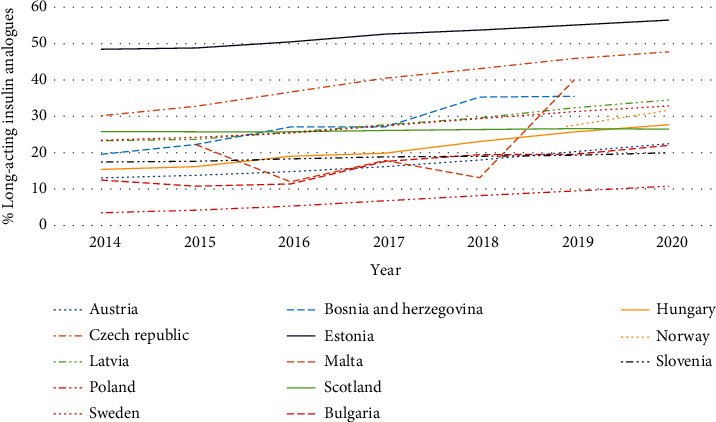
Utilisation of long-acting insulin analogues as a percentage of total insulins over time across Europe (DDD based).

There has been growing utilisation for long-acting insulin analogues over time among both Western and CEE countries, with no obvious difference in the rates of utilisation and increase between Western and CEE countries (Figure 1). This reflects the growing recognition of the role and value of long-acting insulin analogues in the management of patients with diabetes mellitus across Europe coupled with their increasing promotion.

The greatest utilisation of long-acting insulin analogues in recent years was seen in Estonia (56.5% of total insulins), Czech Republic (47.8%), and Malta (40.0%). There was also considerable prescribing of long-acting insulin analogues in Catalonia in recent years reaching 55.2% of total insulins in 2020 (not shown in Figure 1). However, the variable use of long-acting insulin analogues in Malta reflects procurement practices for that year; consequently, rates can be flexible between the years with implications for accuracy for any one year.

The least change in the prescribing patterns for long-acting insulin analogues was seen in Scotland (2.7% increase over time), with the greatest change seen in Poland (210.6% increase over time), but from a low base. In Poland, this may reflect a more cautious attitude towards long-acting insulin analogues coupled with issues of affordability. There was also a more cautious approach to the prescribing of long-acting insulin analogues in Slovenia, with similar prescribing rates over time (10.7% increase between 2014 and 2019). This may again reflect issues of value and affordability; however, more research is needed before we can say anything with certainty.

The stable utilisation of long-acting insulin analogues in Scotland in recent years (Figure 1) may well reflect adherence to the advice from NHS Scotland that patients in Scotland should ideally be started on human intermediate acting insulins, with long-acting insulin analogues only considered based on an assessment of a patient's hypoglycaemic risk. Adherence to agreed guidance is enhanced by regular monitoring of physicians' prescribing of long-acting insulin analogues versus other insulin preparations in Scotland [117]. We have seen monitoring of advice increase adherence rates to prescribed guidance in other disease areas in Scotland [83, 84, 118].

### 3.2. Expenditure for the Different Insulin Preparations Over Time

**Figure 2:**
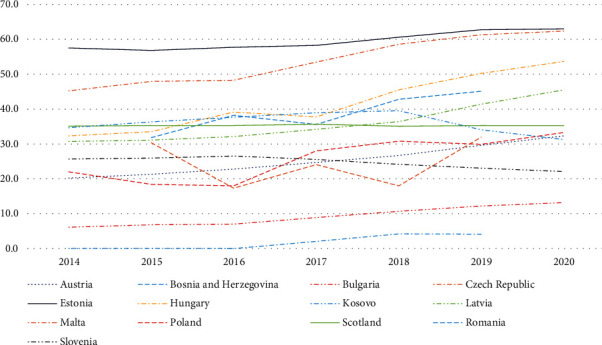
Expenditure on long-acting insulin analogues as a percentage of total insulin expenditure over time among European countries.

The increasing use of long-acting insulin analogues as a percentage of total insulins (Figure 1) was also reflected in similar changes in their expenditure compared with total expenditure on insulins (Figure 2).

Variations ranged from a slight fall in Romania and Slovenia over time with the cost/DDD for originator insulin glargine 100 IU/ml falling by 20.3% over time in Slovenia (Table 1) to a limited change in overall expenditure in Malta with the cost/DDD falling by 61.3% during the study period (Table 1). This compares with an appreciable increase in expenditure of long-acting insulin analogues in Kosovo over time but from a low base.

Increasing expenditure on long-acting insulins in Kosovo in recent years again reflects perceptions of improved patient convenience and outcomes versus standard insulins such as NPH insulins. There is a similar situation in Hungary with expenditure on long-acting insulins reaching 53.7% of total expenditure in recent years, similar to high expenditure rates in Estonia (63% in 2020), the Czech Republic (62.4%), and Latvia (45.5%). There was also appreciable expenditure on long-acting insulin analogues in Catalonia currently at 63.2% of total insulin expenditure (not shown).

The relatively high expenditure on long-acting insulins in Romania in recent years again reflects successful marketing by the originator companies with insulin glargine being one of the top selling medicines in Romania in recent years joined recently by insulin detemir.

### 3.3. Utilisation of Insulin Glargine including Biosimilar 100 IU/ml and 300 IU/ml (Gla-300)

**Figure 3:**
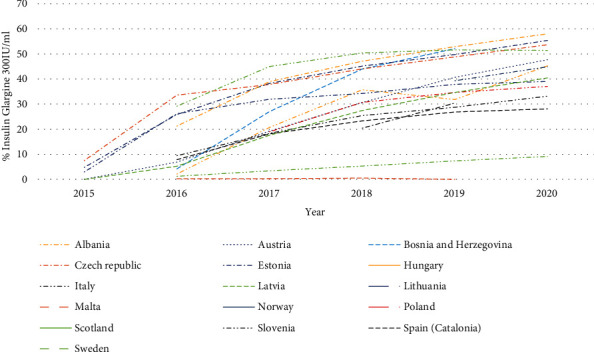
Utilisation of insulin glargine biosimilar (100 IU/ml) as a % of total insulin glargine 100 IU/ml (DDD based) over time across Europe.

There has also been considerable variation in the use of biosimilar insulin glargine (100 IU/ml) versus total insulin glargine across Europe (Figure 3). This reflects a number of differences between countries in terms of switching of prescribing of insulin glargine from 100 IU/ml to patented 300 IU/ml (Gla-300) as well as activities of the originator company to lower its price to make the market less attractive for biosimilars.

**Figure 4:**
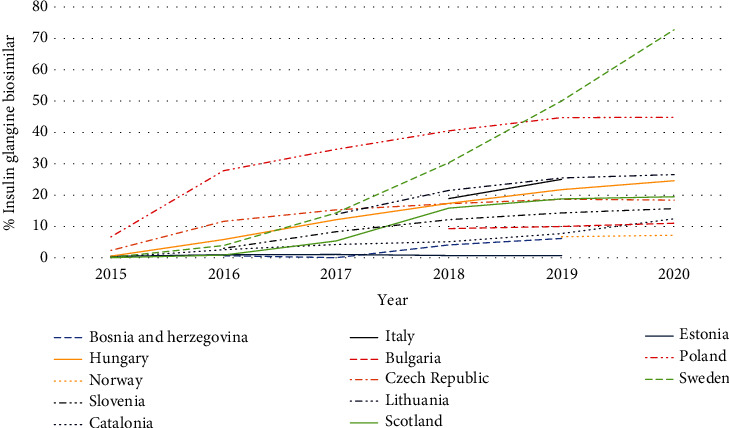
Utilisation of insulin glargine 300 IU/ml (Gla-300) as a % of total insulin glargine (DDD based) across Europe over time

Currently, no biosimilar insulin glargine is marketed in Albania, Austria, or Latvia. This may reflect increasing utilisation of Gla-300 in recent years rising to 45.3%, 47.7%, and 51.4%, respectively, of total insulin glargine in these countries as a result of commercial and other activities (Figure 4). This coupled with reduced prescribing generally of insulin glargine (Latvia), and price reductions of the originator over time (Albania and Latvia) (Table 1) appear to have made the 100 IU/ml biosimilar market unattractive in these countries. This is despite insulin glargine being the predominant long-acting insulin analogue prescribed in Albania in recent years, rising to 81.1% of total long-acting insulin analogues (DDD basis) prescribed.

There is also currently no biosimilar insulin glargine imported into Kosovo due to a number of issues including concerns with their effectiveness and safety versus the originator, and currently there is no biosimilar insulin glargine prescribed in Malta despite very limited use of Gla-300 (Figure 4). This probably reflects the considerable price reduction by the originator company making this market unattractive to biosimilar manufacturers (61.3%, Table 1). Similarly, whilst insulin glargine biosimilar has recently been reimbursed in Romania (Abasaglar® 100), its uptake to date has been very limited (not shown) due to ongoing pricing and reimbursement policies coupled with limited physician incentives to preferentially prescribe biosimilars alongside no copayment issues for patients.

There was also very limited utilisation of insulin glargine biosimilars in Estonia, contrasting with their growing utilisation in Lithuania as another key member of the Baltic States. This again probably reflects the originator company switching promotional activities to patented Gla-300 in Estonia to reduce biosimilar competition, with utilisation of Gla-300 growing to 55.4% of total insulin glargine in 2020 (Figure 4). In addition, the originator company dropping its price by 24.9% over time (Table 1) resulting in limited price differences in recent years between the originator and biosimilars (2.1%-7.1%).

Low and constant utilisation of biosimilar insulin glargine in Bulgaria again reflects continued marketing activities by the originator company coupled with currently a lack of physician incentives to preferentially prescribe biosimilars alongside limited price difference in practice between the originator and the biosimilar (Table 1), with both reducing their prices over time.

Low utilisation of insulin glargine biosimilars in Norway also potentially reflects limited price differences between the originator and biosimilar in recent years (Table 1) coupled with growing utilisation of Gla-300 (Figure 4). This contrasts with Sweden which has the highest biosimilar use among the studied European countries (Figure 3) despite growing use of Gla-300 (Figure 4). This is probably due to a tradition of prescribing of multiple source medicines with compulsory generic substitution in Sweden coupled with ongoing initiatives to enhance the quality and efficiency of prescribing including enhancing the prescribing of biosimilars [55, 106, 119, 120]. Ongoing initiatives also include devolving budgets locally to enhance the focus of ambulatory care physicians on prescribing efficiency.

The situation in Lithuania contrasts with the other Baltic countries as there has been growing utilisation of biosimilar insulin glargine as a percentage of all insulin glargine 100 IU/ml in recent years, reaching 26.5% of total insulin glargine 100 IU/ml in 2020 (Figure 3). This reflects the fact that all long-acting insulin analogues are in the same reference price group with patients covering the additional costs themselves for a more expensive medicine [121, 122]. Having said this, utilisation of the 100 IU/ml formulation has been moderated in recent years in Lithuania by increasing utilisation of Gla-300, rising to 39.0% of all insulin glargine in early 2020 (Figure 4) coupled with price reductions by the originator (21.1% between 2015 and 2020) to limit any copayment differences.

There has also been growing utilisation of insulin glargine biosimilars in Bosnia and Herzegovina (B & H), but from a low base with the state agency recently encouraging physicians to prescribe biosimilars for new patients where possible, with physicians generally following national guidelines in B & H [108, 123]. Greater growth though is hampered by high utilisation of Gla-300, reaching 52.1% of all insulin glargine use in 2019 (Figure 4), and the originator dropping its price to reduce any resultant price differential (Table 1).

The growth in the utilisation of the biosimilar in Hungary is also welcomed as this was not the case with biosimilars for infliximab and rituximab [124, 125]. However, there are now ongoing reforms in Hungary to encourage physicians to start patients on the least expensive biosimilar as well as the reference pricing system with patients required to fund the difference in prices between the originator and any biosimilar themselves [126]. Having said this, utilisation of biosimilar insulin glargine in Hungary is again adversely affected by the originator dropping its price over time (Table 1) coupled with increasing use of Gla-300 reaching 58% of total insulin glargine in recent years (Figure 4).

The growth in the prescribing of biosimilar 100 IU/ml insulin glargine in Italy in recent years (Figure 3) probably reflects ongoing regional and national demand-side measures to enhance the prescribing of biosimilars given some of the price differences seen including for biosimilar insulin glargine (Table 1) and the need to conserve resources [127, 128]. However, greater utilisation of biosimilar insulin glargine may again be hampered by growing utilisation of Gla-300 in Italy in recent years (Figure 4).

We are also seeing growing utilisation of biosimilar insulin glargine in Scotland. However, growth is limited by concerns with switching between the originator and biosimilar 100 IU/ml insulin glargine, with physicians requesting to prescribe by brand name [64, 65]. This works in the UK with community pharmacists not allowed to substitute an originator with a generic without physician approval [80, 129]. Having said this, there are traditionally very high rates of INN prescribing in Scotland [83, 84, 118]. There is currently low use of Gla-300 in Scotland as a result of ongoing prescribing guidance to limit its use enhanced by concerns that patients may inadvertently over dose [86].

The appreciably higher utilisation of biosimilar insulin glargine in Poland in recent years compared with a number of other CEE countries (Figure 3) may well be facilitated by a flat reimbursement rate with patients paying the price difference for a more expensive originator [126, 130]. Alongside this, the Ministry of Health and the National Health Insurance Fund in Poland are both looking to encourage the use of biosimilars to save resources especially as Poland is a leading producer of biosimilars in Europe [130, 131]. However, their prescribing is also hampered by growing utilisation of Gla-300 reaching 37.1% of total insulin glargine by early 2020 (Figure 4).

Prices are also now similar between the biosimilar insulin glargine and the originator in the Czech Republic potentially impacting on its use following a fall in originator prices (25.5%) and also biosimilar prices (7.7%) (Table 1). As a result, there is limited use of biosimilars despite growing utilisation of long-acting insulin analogues in the Czech Republic reaching 47.8% of total insulins in 2020 (Figure 1). There are current restrictions regarding the prescribing of long-acting insulin analogues in the Czech Republic, with long-acting insulin analogues only reimbursed if current treatment regimens fail to achieve target HbA1c levels below 60 mmol/mol or if patients prescribed human insulins repeatedly experience severe hypoglycaemia. Concomitant with this, treatment with long-acting insulin analogues should no longer be reimbursed unless there is a demonstrable improvement in the patient's HbA1c levels within three months of initiation, i.e., a reduction by at least 10%, or significant reduction in the incidence of hypoglycaemia. However, there is currently variable follow-up of these restrictions in practice.

### 3.4. Potential Strategies to Enhance the Prescribing of Biosimilar Insulin Glargine

Box 1 contains a number of potential strategies to enhance the utilisation of biosimilar long-acting insulin analogues in Europe. This builds on currently variable utilisation of biosimilar insulin glargine across Europe. This is seen as essential to stimulate the market for the benefit of key stakeholders in the future.

## 4. Discussion

We believe this is the most comprehensive study to date to explore current utilisation and expenditure patterns for different insulin preparations, with a particular focus on insulin glargine and its biosimilars, across Europe. There has typically been increasing utilisation of long-acting insulin analogues across Europe despite their higher price (Figure 1), reflecting perceived patient benefits in terms or reduced hypoglycaemia and greater convenience. This increased use is seen in both Western European and CEE countries demonstrating that affordability is not an issue unlike a number of lower- and middle-income countries [35, 143, 145]. Similar patterns were seen when evaluating changes in expenditure on long-acting insulin analogues as a percentage of total expenditure on insulins (Figure 2).

However, there are concerns with limited or no use of biosimilar insulin glargine in a number of European countries despite a number of studies showing no difference in effectiveness and safety between the originator and biosimilars [57–60] (Section 3.3). This is due to a number of factors including promotional efforts by the originator company to change prescriptions to patented Gla-300 with limited demand-side initiatives from health authorities to discourage this with the exception of Scotland with its prescribing suggestions to limit the use of Gla-300 [86]. In addition, the company lowering the price of the originator often to near or similar to biosimilar prices (Table 1), which coupled with concerns with different devices between the different insulin glargine 100 IU/ml formulations in some markets, has further limited biosimilar use. Alongside this, the continued domination of the insulin market by three manufacturers discourages competition [36, 46].

These issues need to be addressed to enhance the attractiveness of the biosimilar long-acting insulin analogue market, especially with the potential for low cost of goods [46, 67]. We have seen with biosimilars for managing patients with inflammatory diseases such as rheumatoid arthritis that increased competition can lead to low prices for biosimilars [52, 54, 56, 146], and this should be encouraged for long-acting insulin analogues in Europe. Failure to do so will limit the attractiveness of this market to other manufacturers of biosimilar insulin glargine as well as potential manufacturers of other long-acting insulin analogues as these compounds lose their patents. This will be to the detriment of key stakeholder groups especially given rising rates on diabetes across Europe [9] and growing resource issues post-COVID-19.  Box 1 contains a number of activities that European health authorities can instigate to increase competition and subsequent prescribing of biosimilar long-acting insulin analogues, building on demand-side and other measures in other disease areas, and we will be monitoring these in the future.

We are aware of a number of limitations with this study. These include the fact that we did not include all European countries. However, we do not believe that increasing the number of European countries would have appreciably altered our findings. In addition, we only used health authority and health insurance company databases. This was deliberate for the reasons stated. Thirdly, we used DDDs for documenting and analysing utilisation data aware though of the potential problems with Gla-300. This was again deliberate for the reasons stated. Finally, we did not undertake an in-depth analysis of the rationale behind the trends seen in each country. However, feedback was based on the experience of senior-level coauthors in each country. Consequently, we believe our findings and suggestions are robust providing future direction.

## 5. Conclusion

In conclusion, we have seen growing use of long-acting insulin analogues across Europe reflecting their perceived benefits with improving compliance and reducing hypoglycaemia. However, there are concerns with limited or no use of biosimilars of long-acting insulin analogues in a number of European countries due to a number of factors. These include promotional efforts by the originator company and price reductions matching those of biosimilar manufacturers. These issues need to be addressed to enhance the utilisation of biosimilars in the future to the benefit of all key stakeholder groups.

## Figures and Tables

**Box 1:**
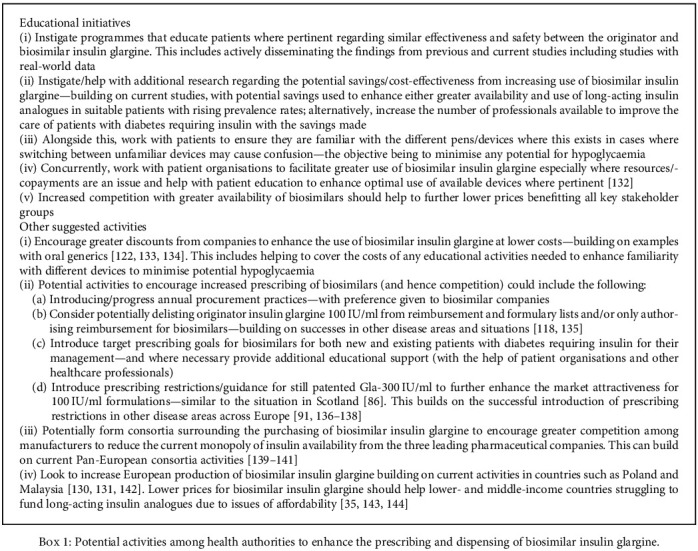
Potential activities among health authorities to enhance the prescribing and dispensing of biosimilar insulin glargine.

**(a):** 

	Albania	Austria	B & H	Bulgaria	Catalonia (Spain)
% difference originator vs. biosimilar price					
Launch of the biosimilar	Not applicable	Not applicable	No difference	4.7%	30.0%
Latest difference	Not applicable	Not applicable	7.9%	5.7%	Similar
% price change over time (from 2014/2015 to 2020)					
Originator	-32.0%	No change	-11.3%	-10.8%	-23.1%
Biosimilar	Not applicable	Not applicable	-17.1%	-11.7%	No change

**(b):** 

	Czech Republic	Estonia	Hungary	Italy	Latvia	Lithuania
% difference originator vs. biosimilar price						
Launch of the biosimilar	17.1%	16.4%	28.2%	Not recorded	Not applicable	12.3%
Latest difference	Similar	7.1%	1.6%	31.6%	Not applicable	Similar
% price change over time (from 2014/2015 to 2020)						
Originator	-25.5%	-24.9%	-21.2%	52.3%	-14.4%	-21.1%
Biosimilar	-7.7%	Stable	1.2%	Not recorded	Not applicable	-6.8%

**(c):** 

	Malta	Norway	Poland	Scotland	Slovenia	Sweden
% difference originator vs. biosimilar price						
Launch of the biosimilar	Not applicable	12.1%	24.7%	18.1%	22.9%	13.6%
Latest difference	Not applicable	5.9%	0.2%	7.5%	9.9%	0.6%
% price change over time (from 2014/2015 to 2020)						
Originator	-61.3%	-3.6%	-31.1%	-9.0%	-20.3%	-12.7%
Biosimilar	Not applicable	2.1%	-6.5%	No change	No change	-1.4%

## Data Availability

The content of health authority and health insurance company databases is typically confidential. However, reasonable requests for information will be considered and actioned where possible. The coauthors from the various European countries have been responsible for the content and accuracy of the information they have provided.
