# Corrigendum to “Utilisation Trend of Long-Acting Insulin Analogues including Biosimilars across Europe: Findings and Implications”

**DOI:** 10.1155/2023/9757348

**Published:** 2023-04-07

**Authors:** Brian Godman, Magdalene Wladysiuk, Stuart McTaggart, Amanj Kurdi, Eleonora Allocati, Mihajlo Jakovljevic, Francis Kalemeera, Iris Hoxha, Anna Nachtnebel, Robert Sauermann, Manfred Hinteregger, Vanda Marković-Peković, Biljana Tubic, Guenka Petrova, Konstantin Tachkov, Juraj Slabý, Radka Nejezchlebova, Iva Selke Krulichová, Ott Laius, Gisbert Selke, Irene Langner, András Harsanyi, András Inotai, Arianit Jakupi, Svens Henkuzens, Kristina Garuolienė, Jolanta Gulbinovič, Patricia Vella Bonanno, Jakub Rutkowski, Skule Ingeberg, Øyvind Melien, Ileana Mardare, Jurij Fürst, Sean MacBride-Stewart, Carol Holmes, Caridad Pontes, Corinne Zara, Marta Turu Pedrola, Mikael Hoffmann, Vasileios Kourafalos, Alice Pisana, Rita Banzi, Stephen Campbell, Bjorn Wettermark

**Affiliations:** ^1^Strathclyde Institute of Pharmacy and Biomedical Sciences, University of Strathclyde, Glasgow G4 0RE, UK; ^2^Division of Public Health Pharmacy and Management, School of Pharmacy, Sefako Makgatho Health Sciences University, Pretoria, South Africa; ^3^School of Pharmaceutical Sciences, Universiti Sains Malaysia, Penang, Malaysia; ^4^Chair of Epidemiology and Preventive Medicine, Medical College, Jagiellonian University, Krakow, Poland; ^5^HTA Consulting, Starowiślna Str. 17/3, 31-038 Krakow, Poland; ^6^Public Health Scotland, Gyle Square, 1 South Gyle Crescent, Edinburgh, UK; ^7^Department of Pharmacology, College of Pharmacy, Hawler Medical University, Erbil, Iraq; ^8^Istituto di Ricerche Farmacologiche “Mario Negri” IRCCS, Milan, Italy; ^9^Institute of Advanced Manufacturing Technologies, Peter the Great St. Petersburg Polytechnic University, St. Petersburg, Russia; ^10^Institute of Comparative Economic Studies, Hosei University Tokyo, Japan; ^11^Department of Global Health Economics and Policy, University of Kragujevac, Serbia; ^12^Department of Pharmacy Practice and Policy, Faculty of Health Sciences, University of Namibia, Windhoek, Namibia; ^13^Department of Pharmacy, Faculty of Medicine, University of Medicine, Tirana, Albania; ^14^Dachverband der Österreichischen Sozialversicherungen, Kundmanngasse 21, AT-1030 Vienna, Austria; ^15^Faculty of Medicine, Department of Social Pharmacy, University of Banja Luka, Banja Luka, Bosnia and Herzegovina; ^16^Faculty of Medicine, Department of Medicinal Chemistry, University of Banja Luka, Banja Luka, Bosnia and Herzegovina; ^17^Agency for Medicinal Product and Medical Devices of Bosnia and Herzegovina, 78000 Banja Luka, Bosnia and Herzegovina; ^18^Faculty of Pharmacy, Department of Social Pharmacy and Pharmacoeconomics, Medical University of Sofia, Sofia, Bulgaria; ^19^State Institute for Drug Control, Prague, Czech Republic; ^20^Department of Medical Biophysics, Faculty of Medicine in Hradec Králové, Charles University, Simkova 870, 500 03 Hradec Králové, Czech Republic; ^21^State Agency of Medicines, Nooruse 1, 50411 Tartu, Estonia; ^22^Wissenschaftliches Institut der AOK (WIdO), Rosenthaler Straße 31, 10178 Berlin, Germany; ^23^Department of Health Policy and Health Economics, Eotvos Lorand University, Budapest, Hungary; ^24^Syreon Research Institute and Semmelweis University, Center of Health Technology Assessment, Budapest, Hungary; ^25^Faculty of Pharmacy, UBT Higher Education Institute, Pristina, Kosovo; ^26^Independent Consultant, Riga, Latvia; ^27^Department of Pathology, Forensic Medicine and Pharmacology, Institute of Biomedical Sciences, Faculty of Medicine, Vilnius University, Vilnius, Lithuania; ^28^Department of Health Services Management, University of Malta, Valletta, Malta; ^29^Medicines Committee, Oslo University Hospitals, Oslo, Norway; ^30^Faculty of Medicine, Public Health and Management Department, “Carol Davila” University of Medicine and Pharmacy Bucharest, 050463 Bucharest, Romania; ^31^Health Insurance Institute, Miklosiceva 24, SI-1507 Ljubljana, Slovenia; ^32^Pharmacy Services, Greater Glasgow and Clyde (NHS GGC), Glasgow, UK; ^33^NHS Lothian, Edinburgh, UK; ^34^Drug Department, Catalan Health Service, Gran Via de les Corts Catalanes, 08007 Barcelona, Spain; ^35^Department of Pharmacology, Therapeutics and Toxicology, Universitat Autònoma de Barcelona, Barcelona, Spain; ^36^NEPI-Nätverk för Läkemedelsepidemiologi, Stockholm, Sweden; ^37^National Organization for the Provision of Healthcare Services (EOPYY), Athens, Greece; ^38^Department of Global Public Health, Karolinska Institutet, Stockholm, Sweden; ^39^Centre for Primary Care and Health Services Research, School of Health Sciences, University of Manchester, Manchester M13 9PL, UK; ^40^NIHR Greater Manchester Patient Safety Translational Research Centre, School of Health Sciences, University of Manchester, Manchester, UK; ^41^Department of Pharmacy, Disciplinary Domain of Medicine and Pharmacy, Uppsala University, Uppsala, Sweden

In the article titled “Utilisation Trend of Long-Acting Insulin Analogues including Biosimilars across Europe: Findings and Implications” [1], the captions of Figures 3 and 4 were incorrect. The corrected captions for Figures 3 and 4 appear below.

## Figures and Tables

**Figure 1:**
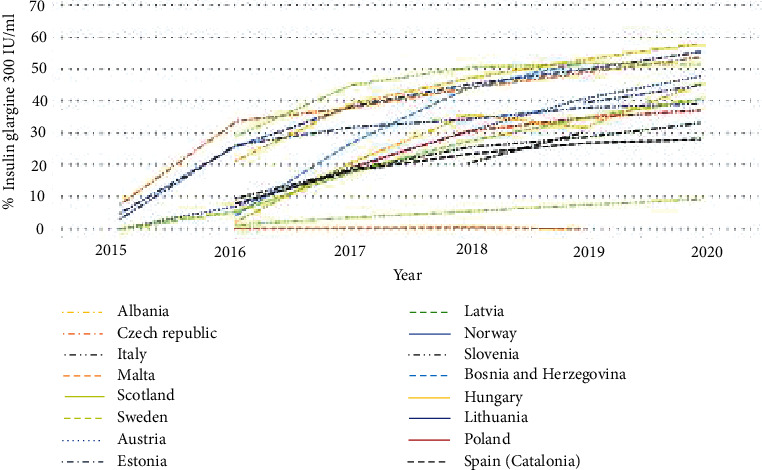
Utilisation of insulin glargine 300 IU/ml (Gla-300) as a % of total insulin glargine (DDD based) across Europe over time.

**Figure 2:**
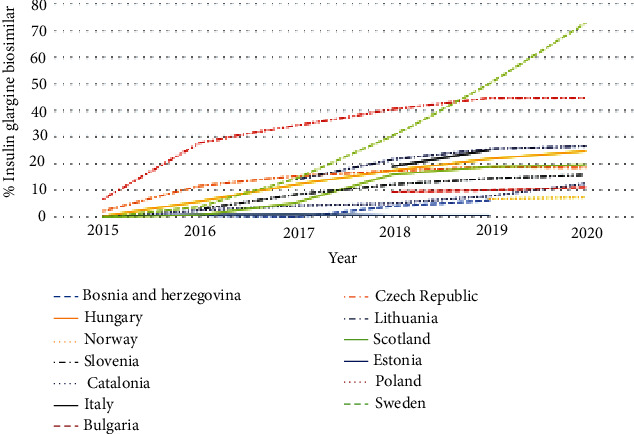
Utilisation of insulin glargine biosimilar (100 IU/ml) as a % of total insulin glargine 100 IU/ml (DDD based) over time across Europe.
